# Carbon Monoxide Poisoning was Associated With Lifetime Suicidal Ideation: Evidence From A Population-Based Cross-Sectional Study in Hebei Province, China

**DOI:** 10.3389/ijph.2022.1604462

**Published:** 2022-06-15

**Authors:** Long Sun, Keqing Li, Yunshu Zhang, Lili Zhang

**Affiliations:** ^1^ Centre for Health Management and Policy Research, School of Public Health, Cheeloo College of Medicine, Shandong University, Jinan, China; ^2^ National Health Commission of China Key Lab for Health Economics and Policy Research (Shandong University), Jinan, China; ^3^ The Sixth People Hospital of Hebei Province, Baoding, China

**Keywords:** China, population-based study, carbon monoxide poisoning, suicidal ideation, suicide plan

## Abstract

**Objectives:** We want to test the association between carbon monoxide poisoning (CMP) experiencing and lifetime suicidal ideation/suicide plan among community residents.

**Methods:** This is a population-based cross-sectional study conducted among community residents in Hebei province, China. We analyzed a total of 21,376 valid questionnaires. CMP experience and lifetime suicidal ideation/suicide plan were assessed in this study. Logistic regression and false discovery rate correction were conducted to analyze the associations and correct the *p* values.

**Results:** We found that CMP (OR = 2.56, *p <* 0.001, corrected-p = 0.001) was associated with lifetime suicidal ideation, and the other risk factors were female (*OR* = 0.53, *p <* 0.001, corrected-p = 0.001). The association between CMP and suicide plan was not supported after false discovery rate correction (*OR* = 2.15, *p* = 0.035, corrected-*p* = 0.385). For the CMP patients, experiencing ≥2 times CMP (*OR* = 2.76, *p* = 0.001, corrected-p = 0.011) was also in higher risk of lifetime suicidal ideation. The association between CMP times and lifetime suicidal plan was not supported after false discovery rate correction (*OR* = 4.95, *p* = 0.021, corrected-*p* = 0.231).

**Conclusion:** CMP patients are in higher risk of lifetime suicidal ideation. For CMP patients, some strategies are needed to control their suicidal ideation.

## Introduction

According to the latest estimates by World Health Organization (WHO), there were more than 700,000 people died by suicide in 2019, which meant that one in 100 deaths was caused by suicide, and the number of death caused by suicide was also higher than HIV, malaria, breast cancer, war or homicide [1]. In China, although the suicide rates decreased in the recent years [2], suicide was also the top 10 causes of death [3]. All of these informed us that suicide was an important public health and society problem in China and some other countries worldwide.

Carbon monoxide poisoning (CMP), mainly caused by accidences [4, 5], was a frequent cause of acute toxicity with high morbidity and mortality worldwide [6, 7]. Previous studies had found that nervous system disorders, including sleep initiation and maintenance disorders, tension-type headache, occur in over half of the patients within the first years after CMP [8]. In recent years, several longitudinal studies also supported that sleep disorders can be warning signs for imminent suicidal thought and behaviors [9–11]. Basing on these longitudinal findings, we have reasons to assume the association between CMP and suicidal thought and behaviors, including suicidal ideation and suicide plan.

In the other sides, the central nervous system was the most frequently affected by CMP, and most of the frequent outcomes of CMP were delayed neuropsychiatric sequelae, such as delayed encephalopathy [12, 13]. Patients with delayed neuropsychiatric sequelae would have changes on individual’s mental status and behaviors [14–16]. As one kind of individual’s behaviors, suicide may also be associated with the changes of mental status and behaviors. Although the mechanism for this relationship was not clear until now [17], cognitive decline may be one of the explanations for this association, which is also a risk factor for suicidal ideation [18–20]. Actually, some publications also reported that there was nucleotide variation in central nervous system genes for suicide risk [21, 22]. In practice, there were also several case studies, which reported several cases conducted suicide after CMP. One was a truck driver, and he committed suicide 15 months later after CMP [23]. Another case was a 12-year-old girl, who also conducted suicide after CMP [24]. However, to our knowledge, this association between CMP and suicidal ideation was not tested in the population level until now.

To fill the gap, we conducted a population-based cross-sectional study to test the association between CMP and suicidal ideation/suicide plan. As two kinds of suicidal behaviors, suicidal ideation and suicide plan had been proved to be an important predictor for the following suicidal behaviors [25, 26], which should be paid more attentions. Establishing the associations between CMP and suicidal ideation/suicide plan is also helpful for us to further understand the suicidal behaviors and the impact of CMP. If the association can be identified, the findings not only remind our attention on the nature and the pathogenesis of CMP, but also draw us the further strategies to control suicidal ideation, especially for the clinicians.

## Methods

### Study Sample and Design

This study was conducted in Chinese Hebei province from June to August 2018 with a cross-sectional design. Hebei located in the north of China, and its Gross Domestic Product (GDP) ranked 9th in all the 31 provinces of Chinese mainland [27]. To make sure the representativeness of the sample, a three-stage multistage stratified cluster sampling was used to select the community residents aged 18 years and above. First, five cities (Shijiazhuang, Baoding, Xingtai, Zhangjiakou, Qinhuangdao) were randomly selected from all the 11 cities in Hebei. Second, we randomly selected three counties (rural region) and one district (urban region) in each selected city. Third, one township or sub-district was randomly selected from each county or district. Fourth, we randomly selected one village (community) in each selected township (sub-district). Totally, we selected 15 rural villages and 5 urban communities to conduct the study’s survey. Residents aged 18 years and above in the selected villages or communities were interviewed in this study. Finally, 21,376 valid questionnaires were analyzed to get the association between carbon monoxide poisoning and suicidal ideation.

### Interviewing Procedures

Before the survey, the research group trained all the interviewers for 2 days to make sure they had fully understood the research and questionnaire. After the training, a face-to-face interview was scheduled by one interviewer for all the participants upon their agreement with written informed consent form. To ensure the quality of the questionnaire, all the questionnaires were checked by other reviewer on each interview day. For the questionnaires with missing data or logical problems, the interviewers were asked to revisit or call the interviewees on the next day. The study protocol was approved by the Institutional Review Board (IRB) of Hebei Mental Health Center before data collection. Written informed consent was obtained from all the participants.

### Measures

#### Lifetime Suicidal Ideation and Plan

Lifetime suicidal ideation was assessed by the question that “whether you ever seriously thought about killing yourself?” The answer can be chosen from yes (1) or no (0). For the people with positive answers, they would be asked that whether they ever made a plan for suicide. Participants who chose yes were seen as experiencing suicidal ideation or plan. These questions were used to assess suicidal ideation and plan in many previous studies, such as United States National Comorbidity Survey (NCS), National Comorbidity Survey Replication (NCSR) [28] and many other studies worldwide [29, 30].

#### Lifetime Carbon Monoxide Poisoning Experience and Its Times

Lifetime carbon monoxide poisoning (CMP) experience was evaluated by the question that “have you ever experienced carbon monoxide poisoning?” The answers were also yes (1) and no (0). Subjects who answered yes were analyzed as experiencing CMP. For people who experienced CMP, we further asked the times for their CMP experiencing. As there were few subjects experienced CMP more than 2 times, we recoded it into 1 time (0) and ≥2 times (1).

#### Social-Demographic Variables

Gender was evaluated by male (1) or female (0). Age was calculated by the participants’ date of birth. Ethnicity was evaluated by Han (1) and others (0). Education level was assessed by the participants’ academic degree with the 4 options, less of elementary (1), elementary school (2), middle school (3) and high school or above (4). Married status was evaluated through one question about the participants’ married status. The answers were never married, married, divorced, widowed, deuterogamist, and others. As the small percentage of the last four answers, we recoded it into unmarried (1), married (2), and others (3). Region was assessed by asking the region where the participants lived, and the answers contained urban (0) and rural (1). Living alone was evaluated by number of people lived with the participants. For participants lived without other persons were recoded into living alone (1), and others were recoded into not living alone (0).

### Statistical Methods

SPSS for Windows 24.0 (web version) and R (version 4.2.0) were used to conduct the data analysis. Student’s t-test or Chi-square test was performed to compare the means or proportions between social-demographic variables, CMP experiencing (times), and suicidal ideation/suicide plan. Logistic regression was performed to examine the association between CMP experiencing (times) and suicidal ideation/suicide plan after controlling social-demographic variables. False discovery rate correction with Benjamini-Hochberg method was further performed to correct the *p* values of logistic regressions. All significance tests were two-tailed, and a *p*-value of 0.05 or lower was considered as statistical significance.

## Results

In this study, we interviewed a total of 21,376 participants aged 18 years and above among community residents in Hebei province, China. The prevalence of lifetime suicidal ideation and suicide plan was 1.4% (289/21,376) and 0.3% (67/21,376), respectively. In Table 1, we descripted the sample characteristics. In this sample, there were 11,537 females (54.0%) and 9,839 males (46.0%), and the mean age was 50.85 years. The detailed information could be found in the second column of Table 1. We also analyzed the factors associated with lifetime suicidal ideation, and the risk factors were female ( $\chi^{2}$ = 33.93, *p <* 0.001), older age (*t =* 5.51, *p <* 0.001), lower education level ( ${\text{χ}}^{2}$ = 33.57, *p* < 0.001), ever married ( $\chi^{2}$ = 48.09, *p <* 0.001), living alone ( $\chi^{2}$ = 31.84, *p <* 0.001) and CMP experience ( $\chi^{2}$ = 49.40, *p <* 0.001). For lifetime suicide plan, the risk factors were older age (*t* = 2.20, *p <* 0.05), ever married ( $\chi^{2}$ = 6.35, *p <* 0.05) and CMP experience ( $\chi^{2}$ = 5.81, *p <* 0.05). The detailed information could be found in Table 1.

**TABLE 1:** Sample description and single analysis between social-demographic variables, carbon monoxide poisoning and suicidal ideation/suicide plan (Hebei, China. 2018).

Variables	Total	Suicidal ideation		*t/* $\chi^{2}$	Suicide plan		*t/* $\chi^{2}$
		Yes, n (%)	No, n (%)		Yes, n (%)	No, n (%)	
All	21,376 (100.0)	289 (1.4)	21,087 (98.6)	—	67 (0.3)	21,309 (99.7)	—
Gender				33.93***			1.41
Male	9,839 (46.0)	84 (0.9)	9,755 (99.1)		26 (0.3)	9,813 (99.7)	
Female	11,537 (54.0)	205 (1.8)	11,332 (98.2)		41 (0.4)	11,496 (99.6)	
Age (yr, mean ± SD)	50.85 ± 16.30	56.10 ± 14.50	50.78 ± 16.31	5.51***	56.22 ± 14.48	50.84 ± 16.30	2.20*
Ethnicity				0.34			2.36
Hans	20,094 (94.0)	274 (1.4)	19,820 (98.6)		60 (0.3)	20,034 (99.7)	
Others	1,282 (6.0)	15 (1.2)	1,267 (98.8)		7 (0.5)	1,275 (99.5)	
Education				33.57***			7.10
Less than elementary	2,691 (12.6)	55 (2.0)	2,636 (98.0)		9 (0.3)	2,682 (99.7)	
Elementary	5,264 (24.6)	96 (1.8)	5,168 (98.2)		25 (0.5)	5,239 (99.5)	
Middle school	8,274 (38.7)	99 (1.2)	8,175 (98.8)		23 (0.3)	8,251 (99.7)	
High school or above	5,147 (24.1)	39 (0.8)	5,108 (99.2)		10 (0.2)	5,137 (99.8)	
Married Status				48.09***			6.35*
Unmarried	1,548 (7.2)	13 (0.8)	1,535 (99.2)		3 (0.2)	1,545 (99.8)	
Married	18,487 (86.5)	230 (1.2)	18,257 (98.8)		55 (0.3)	18,432 (99.7)	
Others	1,341 (6.3)	46 (3.4)	1,295 (96.6)		9 (0.7)	1,332 (99.3)	
Region				1.55			1.31
Urban	5,100 (23.9)	60 (1.2)	5,040 (98.8)		12 (0.2)	5,088 (99.8)	
Rural	16,276 (76.1)	229 (1.4)	16,047 (98.6)		55 (0.3)	16,221 (99.7)	
Living alone				31.84***			1.45
Yes	1,193 (94.4)	38 (3.2)	1,155 (96.8)		6 (0.5)	1,187 (99.5)	
No	20,183 (5.6)	251 (1.2)	19,932 (98.8)		61 (0.3)	20,122 (99.7)	
CMP				49.40***			5.81*
Yes	1345 (6.3)	47 (3.5)	1,298 (96.5)		9 (0.7)	1,336 (99.3)	
No	20031 (93.7)	242 (1.2)	19,789 (98.8)		58 (0.3)	19,973 (99.7)	

Note: SD, means standard error; CMP, means carbon monoxide poisoning. *, *p* < 0.05; **, *p* < 0.01; ***, *p* < 0.001.

In Table 2, logistic regressions were conducted to analyze the factors associated with lifetime suicidal ideation/suicide plan, and false discovery rate correction with Benjamini-Hochberg method was also performed to correct the *p* values. The results of logistic regression supported that the risk factors of lifetime suicidal ideation were female (*OR* = 0.54, *p <* 0.001), older age (*OR* = 1.01, *p* = 0.029), elementary education (*OR* = 1.77, *p* = 0.007), not being married (*OR* = 0.59, *p* = 0.015), and CMP experience (*OR* = 2.56, *p <* 0.001). After the false discovery rate correction, female (corrected-*p* = 0.001), elementary education (corrected-*p* = 0.026), not being married (corrected-*p* = 0.041), and CMP experience (corrected-*p* = 0.001) were positively associated with lifetime suicidal ideation. For lifetime suicide plan, CMP experience (*OR* = 2.15, *p* = 0.035) was the only risk factor associated with suicide plan. After false discovery rate correction, the association between CMP experience and suicide plan disappeared (corrected-*p* = 0.385). The detailed information can be found in Table 2.

**TABLE 2:** Logistic regressions for the associations between social-demographic variables, carbon monoxide poisoning, and suicidal ideation/suicide plan (Hebei, China. 2018. *n* = 21,376).

Variables	Suicidal ideation			Suicide plan		
	OR (95%CI)	p	Corrected-p	OR (95%CI)	p	Corrected-p
Male	0.53 (0.41, 0.69)	<0.001	0.001	0.79 (0.47, 1.31)	0.353	0.527
Age	1.01 (1.00, 1.02)	0.029	0.064	1.01 (0.99, 1.03)	0.261	0.527
Hans	1.15 (0.68, 1.95)	0.606	0.606	0.54 (0.25, 1.20)	0.129	0.527
Education (Ref. = High school or above)						
Less than elementary	1.44 (0.89, 2.34)	0.141	0.194	0.95 (0.34, 2.67)	0.923	0.941
Elementary	1.77 (1.17, 2.69)	0.007	0.026	1.72 (0.76, 3.89)	0.197	0.527
Middle school	1.46 (0.99, 2.16)	0.054	0.099	1.24 (0.57, 2.69)	0.583	0.713
Married Status (Ref. = Others)						
Unmarried	0.66 (0.32, 1.34)	0.247	0.302	0.52 (0.12, 2.29)	0.383	0.527
Married	0.59 (0.38, 0.90)	0.015	0.041	0.53 (0.22, 1.29)	0.162	0.527
Rural region	1.14 (0.84, 1.55)	0.416	0.458	1.36 (0.70, 2.64)	0.370	0.527
Living alone	1.44 (0.91, 2.27)	0.118	0.185	0.96 (0.34, 2.73)	0.941	0.941
CMP	2.56 (1.86, 3.53)	<0.001	0.001	2.15 (1.06, 4.37)	0.035	0.385
Constant	0.008	<0.001	—	0.004	<0.001	—
R^2^	0.042	—	—	0.021	—	—

Note: OR, means odd ratio; CI, means confidence interval; CMP, means carbon monoxide poisoning.

As the findings about the significant association between CMP and lifetime suicidal ideation, we further analyzed the association between CMP times and lifetime suicidal ideation/suicide plan. In the whole sample, there were 6.3% (1,345/21,376) of the participants experienced CMP in their lives. We firstly analyzed the sample characteristics for the people who ever experienced CMP, which was shown in the second column of Table 3. For people who ever experienced CMP, there were 19.2% (258/1345) of them experienced CMP 2 or more times. The results showed that CMP times were positively associated with lifetime suicidal ideation ( $\chi^{2}$ = 14.18, *p <* 0.001) and suicide plan ( $\chi^{2}$ = 7.73, *p <* 0.01). The detailed information was shown in Table 3.

**TABLE 3:** Sample description and single analysis between social-demographic variables, carbon monoxide poisoning times and suicidal ideation/suicide plan among people experienced carbon monoxide poisoning (Hebei, China. 2018).

Variables	Total	Suicidal ideation		t/ $\chi^{2}$	Suicide plan		t/ $\chi^{2}$
		Yes, n (%)	No, n (%)		Yes, n (%)	No, n (%)	
All	1,345 (100.0)	47 (3.5)	1,298 (96.5)	—	9 (0.7)	1336 (99.3)	—
Gender				8.05**			1.77
Male	426 (31.7)	6 (1.4)	420 (98.6)		1 (0.2)	426 (99.8)	
Female	919 (68.3)	41 (4.5)	878 (95.5)		8 (0.9)	911 (99.1)	
Age (yr, mean ± SD)	55.03 ± 13.70	55.53 ± 14.06	55.01 ± 13.69	0.26	47.44 ± 14.04	55.08 ± 13.69	−1.67
Ethnicity				0.35			6.39*
Hans	1,283 (95.4)	44 (3.4)	1239 (96.6)		7 (0.5)	1276 (99.5)	
Others	62 (4.6)	3 (4.8)	59 (95.2)		2 (3.2)	60 (96.8)	
Education				3.22			2.55
Less than elementary	233 (17.3)	12 (5.2)	221 (94.8)		2 (0.9)	231 (99.1)	
Elementary	375 (27.9)	13 (3.5)	362 (96.5)		1 (0.3)	374 (99.7)	
Middle school	466 (34.6)	16 (3.4)	450 (96.6)		5 (1.1)	461 (98.9)	
High school or above	271 (20.1)	6 (2.2)	265 (97.8)		1 (0.4)	270 (99.6)	
Married Status				6.07*			0.45
Unmarried	47 (3.5)	2 (4.3)	45 (95.7)		0 (0.0)	47 (100.0)	
Married	1,194 (88.8)	37 (3.1)	1,157 (96.9)		8 (0.7)	1186 (99.3)	
Others	104 (7.7)	8 (7.7)	96 (92.3)		1 (1.0)	103 (99.0)	
Region				2.39			0.10
Urban	361 (26.8)	8 (2.2)	353 (97.8)		2 (0.6)	359 (99.4)	
Rural	984 (73.2)	39 (4.0)	945 (96.0)		7 (0.7)	977 (99.3)	
Living alone				13.85***			0.01
Yes	84 (6.2)	9 (10.7)	75 (89.3)		0 (0.0)	84 (100.0)	
No	1261 (93.8)	38 (3.0)	1223 (97.0)		9 (0.7)	1252 (99.3)	
CMP times				14.18***			7.73**
1-time	1,087 (80.8)	28 (2.6)	1,059 (97.4)		4 (0.4)	1083 (99.6)	
≥2 times	258 (19.2)	19 (7.4)	239 (92.6)		5 (1.9)	253 (98.1)	

Note: SD, means standard error; CMP, means carbon monoxide poisoning. *, *p* < 0.05; **, *p* < 0.01; ***, *p* < 0.001.

In Table 4, logistic regressions were also conducted to analyze the association between CMP times and lifetime suicidal ideation/suicide plan among people ever experienced CMP. The results showed that female (*OR* = 0.35, *p* = 0.022), living alone (*O*R = 3.90, *p* = 0.014), and more CMP times (*OR* = 2.76, *p* = 0.001) was positively associated with lifetime suicidal ideation. After false discovery rate correction, only more CMP times (corrected-p = 0.011) was positively associated with lifetime suicidal ideation. For lifetime suicide plan, we found that the risk factors were more CMP times (*OR* = 4.95, *p* = 0.021). The association between CMP times and lifetime suicidal plan was not supported after false discovery rate correction (corrected-*p* = 0.231). The detailed information was shown in Table 4.

**TABLE 4:** Logistic regressions for the associations between social-demographic variables, carbon monoxide poisoning times, and suicidal ideation/suicide plan among people experienced carbon monoxide poisoning (Hebei, China. 2018. *n* = 1,345).

Variables	Suicidal ideation			Suicide plan		
	OR (95%CI)	P	Corrected-p	OR (95%CI)	p	Corrected-p
Male	0.35 (0.14, 0.86)	0.022	0.080	0.40 (0.05, 3.34)	0.394	0.619
Age	0.99 (0.96, 1.02)	0.462	0.895	0.95 (0.89, 1.01)	0.083	0.304
Hans	0.79 (0.23, 2.69)	0.702	0.895	0.21 (0.04, 1.13)	0.069	0.304
Education (Ref. = High school or above)						
Less than elementary	1.28 (0.40, 4.07)	0.678	0.895	3.78 (0.20, 70.51)	0.373	0.619
Elementary	1.04 (0.35, 3.06)	0.945	0.945	0.81 (0.04, 16.95)	0.893	0.997
Middle school	1.22 (0.45, 3.27)	0.698	0.895	2.77 (0.27, 28.27)	0.390	0.619
Married Status (Ref. = Others)						
Unmarried	0.92 (0.15, 5.56)	0.924	0.945	<0.01 (<0.01, —)	0.997	0.997
Married	0.83 (0.28, 2.47)	0.732	0.895	0.25 (0.03, 2.33)	0.223	0.613
Rural region	1.73 (0.75, 3.99)	0.198	0.545	0.93 (0.16, 5.54)	0.932	0.997
Living alone	3.90 (1.32, 11.48)	0.014	0.077	<0.01 (<0.01, —)	0.997	0.997
CMP ≥2 times (Ref. = 1-time CMP)	2.76 (1.50, 5.09)	0.001	0.011	4.95 (1.27, 19.33)	0.021	0.231
Constant	0.04	0.007	—	0.63	0.837	—
R^2^	0.09	—	—	0.18	—	—

Note: OR means odd ratio; CI means confidence interval; CMP means carbon monoxide poisoning.

## Discussion

In this study, we mainly want to analyze the relationship between CMP and suicidal ideation/suicide plan, and the results supported the associations between CMP and suicidal ideation based on a population-based cross-sectional design. The associations between CMP times and suicidal ideation were also analyzed in this study, and the results supported that people ever experienced two or more times CMP were in higher risk of suicidal ideation, comparing with subjects experienced only 1-time CMP. Our results also found that CMP experience and more CMP times were positively associated with suicide plan, but both the associations would be disappeared after false discovery rate correction.

The first aim for this study was to explore the association between CMP and suicidal ideation/suicide plan, and we found that people who experienced CMP were in higher risk of suicidal ideation. To our knowledge, this is the first population study, which reported this relationship. One of the explanations for this relationship was about the neuropsychiatric sequelae after CMP. As we introduced in the Introduction section, people who experienced CMP may have several neuropsychiatric sequelae, such as sleep problems, cognitive decline, chronic pain, and so on [8, 31]. All of these sequelae of CMP were risk factors for suicidal ideation, which had been identified in previous studies [32, 33]. The association between CMP and suicide plan was not supported after false discovery rate correction. It may be explained by the small sample of suicide planner experienced CMP. In this study, although we interviewed more than 20,000 residents, only 9 of them experienced CMP had made suicide plans.

The other finding was about the association between CMP times and suicidal ideation/suicide plan among participants who experienced CMP. Our results supported that people who experienced two or more times CMP were in higher risk of suicidal ideation, and the association between CMP times and suicide plan was not supported after false discovery rate correction. These findings further implied us the associations between CMP and suicidal ideation. Similar to the association between CMP experience and suicidal ideation. The reasons also can be explained by the sequelae of CMP. For people who experienced more times CMP, they might suffer more severe and frequent sequelae of CMP, which were risk factors for suicidal ideation. For the association between CMP times and suicide plan, our results did not support the association between more CMP times and suicide plan after false discovery rate correction. It also could be explained by the small sample of suicide planner experienced CMP. In this study, only 5 residences experienced 2 or more times of CMP.

In this study, the prevalence of suicidal ideation and suicide plan was 1.4% and 0.3%, respectively. For suicidal ideation, the prevalence was varied from 1.1% to 22.5% in different studies [34–36]. The prevalence of suicide plan was also ranged from 0.7% to 15.6% [37–39]. Our results (1.4% and 0.3%) are lower than the previous findings. It may be caused by the regions of the research. Most the publication about Chinese suicidal ideation was interviewed in Chinese metropolitan [26, 40, 41], and our study was conducted in general urban and rural regions. People live in Chinese metropolitan have higher work stress, and they may report higher suicidal ideation than others [42–44]. The other reason may be caused by the decreased suicide rates in China, especially in rural China [45]. For the prevalence of CMP, to our knowledge, there is no population study, which reported this prevalence among community residents, and we cannot compare it with other studies.

In this study, we also found some other risk factors associated with suicidal ideation, such as male, lower education. Actually, all of these control variables have been identified in previous studies. For the gender differences, females are in higher risk of suicidal ideation worldwide [39, 46], but they are in lower risk of suicide death in many countries except China and a few countries [1, 47]. The positive associations between lower education and suicidal ideation/suicide plan were also proved in many studies [11, 29, 48–50].

There are some limitations, which should be considered when we explain the findings. First, this is a cross-sectional study, and we did not interview the temporal orders of CMP and suicidal ideation. Both of them resulted we could not get any causal relationships for the association between CMP and suicidal ideation. Second, the lifetime CMP experience, lifetime suicidal ideation and other control variables were evaluated by the participants’ self-report, and the recall bias cannot be avoided. Third, as we know, CMP is also a method for suicidal behaviors. Although the percentage of suicide by CMP is in a very low level [51, 52], it may also cause some bias for the findings in this study because of the hard works about distinguishing the suicide ideators or planers from suicide attempters using CMP in this study. Forth, this study did not test the inter-rater agreement, and it may also cause some bias for the findings in this study.

Despite these limitations, this is the first population study, which reported the association between CMP, suicidal ideation and suicide plan. The results also remind us that experiencing of CMP can increase the risk of suicidal ideation, and it also implies the effect of CMP on individuals’ behaviors, which can be explored in the future studies. For people experienced CMP, close family and psychiatric follow-up are important and needed to control their suicidal ideation.
